# 2-(2-Pyridylamino)pyridinium tetra­chlorido­zincate(II)

**DOI:** 10.1107/S1600536808007745

**Published:** 2008-05-07

**Authors:** Diego Venegas-Yazigi, Carolina Castillo, Verónica Paredes-García, Andrés Vega, Evgenia Spodine

**Affiliations:** a Facultad de Química y Biología, Universidad de Santiago de Chile, Casilla 40, Correo 33, Santiago, Chile; b Centro para la Investigación Interdisciplinaria, Avanzada en Ciencias de los Materiales, CIMAT, Universidad de Chile, Santiago, Chile; c Departamento de Química, Universidad Tecnológica Metropolitana, Santiago, Chile; d Departamento de Ciencias Químicas, Facultad de Ecología y Recursos Naturales, Universidad Andrés Bello, Av. República 275,3^er^ piso, Santiago, Chile; e Facultad de Ciencias Químicas y Farmaceúticas, Universidad de Chile, Casilla 233, Santiago, Chile

## Abstract

The structure of the title compound, (C_10_H_10_N_3_)_2_[ZnCl_4_], is composed of C_10_H_9_N_3_H^+^ (DPAH^+^) cations and [ZnCl_4_]^2−^ anions. The two pyridyl rings of DPAH^+^ are approximately coplanar, with a dihedral angle of 7.2 (2)° between their corresponding least-squares planes. The proton is disordered in a one-to-one ratio over the two chemically equivalent pyridyl N atoms. An intra­molecular hydrogen bond is formed between the pyridinium H atom and the pyridyl N atom of the other pyridyl ring. The Zn atom lies on a twofold rotation axis. There are also some weak N—H⋯Cl hydrogen bonds. These inter­actions lead to the formation of an alternating zigzag chain in the solid state. The results clearly show that reducing agents normally used in hydro­thermal syntheses, such as metallic zinc employed here, are also active in terms of coordination chemistry.

## Related literature

For related literature, see: Bock *et al.* (1998 ▶); Bose *et al.* (2004 ▶); Camus *et al.* (2000 ▶); Chowdhury *et al.* (2005 ▶); Du & Zhao (2004 ▶); Gillon *et al.* (2000 ▶); Marinescu *et al.* (2005 ▶); Rahaman *et al.* (2005 ▶); Rice *et al.* (2002 ▶); Visser *et al.* (1997 ▶); Willett (1995 ▶); Youngme *et al.* (2005 ▶).
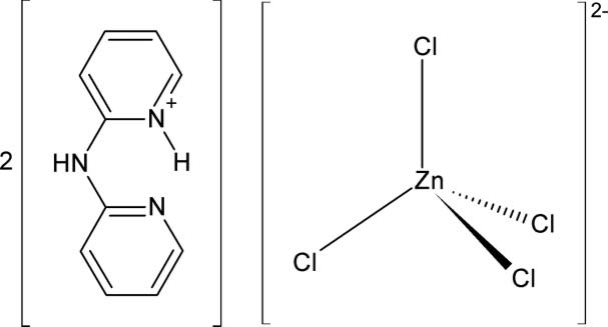


## Experimental

### 

#### Crystal data

(C_10_H_10_N_3_)_2_[ZnCl_4_]
*M* *_r_* = 551.61
Monoclinic, 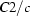

*a* = 14.620 (3) Å
*b* = 11.260 (2) Å
*c* = 14.765 (3) Å
β = 101.13 (3)°
*V* = 2384.9 (8) Å^3^
*Z* = 4
Mo *K*α radiation
μ = 1.50 mm^−1^
*T* = 150 (2) K
0.1 × 0.1 × 0.1 mm

#### Data collection

Siemens SMART CCD area-detector diffractometer
Absorption correction: multi-scan (*SADABS* in *SAINT-NT*; Bruker, 1999 ▶) *T*_min_ = 0.861, *T*_max_ = 0.861
8907 measured reflections
2718 independent reflections
2063 reflections with *I* > 2σ(*I*)
*R*_int_ = 0.029

#### Refinement

*R*[*F* ^2^ > 2σ(*F* ^2^)] = 0.031
*wR*(*F* ^2^) = 0.079
*S* = 1.17
2718 reflections
145 parameters
H atoms treated by a mixture of independent and constrained refinement
Δρ_max_ = 0.53 e Å^−3^
Δρ_min_ = −0.31 e Å^−3^

### 

Data collection: *SMART-NT* (Bruker, 2001 ▶); cell refinement: *SAINT-NT* (Bruker, 1999 ▶); data reduction: *SAINT-NT*; program(s) used to solve structure: *SHELXTL-NT* (Sheldrick, 2008 ▶); program(s) used to refine structure: *SHELXTL-NT*; molecular graphics: *SHELXTL-NT*; software used to prepare material for publication: *SHELXTL-NT*.

## Supplementary Material

Crystal structure: contains datablocks I, New_Global_Publ_Block. DOI: 10.1107/S1600536808007745/zl2105sup1.cif

Structure factors: contains datablocks I. DOI: 10.1107/S1600536808007745/zl2105Isup2.hkl

Additional supplementary materials:  crystallographic information; 3D view; checkCIF report

## Figures and Tables

**Table 1:** Hydrogen-bond geometry (Å, °)

*D*—H⋯*A*	*D*—H	H⋯*A*	*D*⋯*A*	*D*—H⋯*A*
N1—H1N⋯N3	1.06	1.82	2.612 (3)	129
N3—H3N⋯N1	1.03	1.83	2.612 (3)	130
N1—H1N⋯Cl2	1.06	2.73	3.548 (2)	134
N3—H3N⋯Cl2	1.03	2.74	3.477 (3)	129
N2—H2N⋯Cl1^i^	0.93	2.39	3.313 (3)	170

Symmetry code: (i) 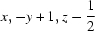.

**Fig. 1.**
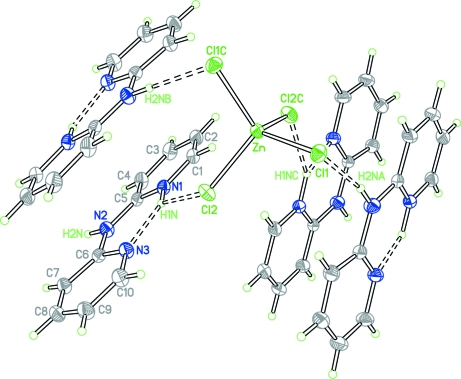
Molecular drawing for I showing hydrogen bonding between [ZnCl4]2-and the organic cation. Atom numbering scheme is included. Displacement ellipsoids at 20%. The second hydrogen position H3N is not included in the diagram for clarity. Symmetry codes: A: x, 1 - y, 1/2 + z; B: 1 - x,1 - y,1 - z; C 1-x. y,3/2 - z.

**Fig. 2.**
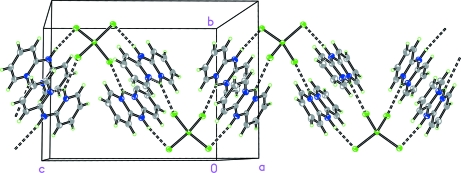
Packing diagram showing the zigzag chains defined by weak intermolecular interactions in the solid state.

## supplementary crystallographic information

### Comment

Core to the crystal engineering of supramolecular compounds are the different strategies that are used to control the molecular aggregation processes. For example, the selection of metal ions that promote the formation of extended arrays, and organic molecules capable of forming hydrogen bonding or close packed structures are fundamental to control the molecular aggregation (Guillon *et al.*, 2000, Rice *et al.*, 2002). Therefore, the strategies adopted in the preparation of crystal structures involve two main concepts. One of them is related with the use of the shape-controlled close packing of molecules, and the second one with the use of the specific interactions to control aggregation of molecular species (Guillon *et al.*, 2000).

2,2-Dipyridylamine (DPA) is used as an organic linker presenting different coordination modes ranging from monodentate to chelating bidentate or bridging tridentate. Furthermore, this organic ligand contains the amino group and aromatic rings that may induce intermolecular hydrogen bonding and π-π stacking interactions that may lead to different structures. (Camus, *et al. *2000, Bose *et al.*, 2004, Rahaman *et al.*2005, Chowdhury *et al.*, 2005, Youmgme *et al.*, 2005, Marinescu *et al.*, 2005).

In this work we inform on the synthesis of a novel crystal structure with a tetrachlorozincate anion obtained by hydrothermal synthesis. The reaction conditions described in the experimental section lead to the oxidation of the metallic zinc used as the reducing agent and, to the formation of a crystalline tetrahalide species, which is present together with the cationic counterion, DPAH^+^.

The organic cation (DPAH^+^) exhibits two pyridinium rings in a highly planar arrangement with a dihedral angle between them of only 7.2 (2)°. This is consistent with the value observed for the isostructural cobalt derivative (DPAH^+^)_2_[CoCl_4_]^2-^ of the title compound (Visser *et al.*, 1997); both compounds being isostructural. The pyridinium nitrogen atoms on the cation are in a "face to face" (or *U*) arrangement, allowing the existence of an intramolecular hydrogen bond (see hydrogen bonding table), also observed for other 2-(pyridin-2-ylamino)pyridinium salts such as the Cl^-^, squarate (C_4_O_4_H)^-^, tetraphenylborate (B(C_6_H_5_)_4_)^-^ (Bock *et al.*, 1998) or NO_3_^-^ (Du & Zhao, 2004) compounds. The parameters for this interaction within these salts are basically identical. A different situation is observed for (DPAH^2+^)[CuCl_4_]^2-^ (Willett, 1995) and (DPAH^2+^)(CF_3_SO_3_)^-^ (Bock *et al.*, 1998) where the molecule is diprotonated, leading to an *S*-like conformation which precludes intramolecular hydrogen bonding.

As expected, the tetrachlorozincate anion, with the zinc atom lying on the twofold axis, displays an almost perfect tetrahedral coordination environment. The [ZnCl_4_]^2-^anion interacts with the cations through weak hydrogen bonds between Cl1 and Cl2 with the DPAH^+^ cations, as summarized in the hydrogen bonding table and depicted in Figure 1. Each tetrachlorozincate anion is bonded in this way to four cations. This differs from what is observed in (DPAH^2+^)[CuCl_4_]^2-^, where the values N(amine)···Cl = 2.133 Å, N(pyridinium)···Cl = 2.307, 2.470 Å) suggest stronger interactions. The rather strong distortion from square planar geometry in this latter molecule has been addressed to the hydrogen bonding interactions (Willett, 1995).

As can be seen in Figure 1, there are two pairs of strictly parallel cations, which are separated by approximately 3.3 Å, a value that is in the range for π-π interactions (Marinescu *et al.*, 2005), but somewhat shorter than the 3.534 (5) Å reported for (DPAH^+^)NO_3_^-^(Du & Zhao, 2004). Each pair of DPAH^+^cations is in connected with a neighboring pair in a "head to tail" fashion (see Figure 1), thus leading to a packing arrangement with a zigzag chain with alternating organic cations and tetrachlorozincate anions as seen in Figure 2. These chains interact in the solid by means of π-π contacts.

### Experimental

The compound was obtained by hydrothermal synthesis employing the following reagents: CrCl_3_.6H_2_O, DPA, V_2_O_5_, Zn, H_3_PO_4 _(85%) and H_2_O (all Aldrich, used without further purification) in the following quantities: 0.1523 g, 0.28 g, 0.1438 g, 0.1054 g, 0.58 ml and 5 ml respectively. This mixture was sealed in a 23 ml PTFE-lined stainless steel autoclave, and heated to 393 K (120°C) for 72 h. The yield of the studied compound was approximately  10%, and X-ray quality crystals were directly selected from the bulk mixture of products.

### Refinement

The hydrogen atoms positions were calculated after each cycle of refinement with *SHELXL* (Bruker,1999) using a riding model with C—H distance equal to 0.96 Å. *U*_iso_(H) values were set equal to 1.2*U*_eq_ of the parent carbon atom. At the final stages of refinement, the hydrogen bonded to the nitrogen atoms becomes evident in the Fourier Difference Map, with some disorder. This was modeled considering two half-occupied hydrogen sites, one on each pyridyl nitrogen atom. These N—H hydrogen atoms were then refined using a riding model with C—N—H angles idealized for amide H atoms, but the the N—H distances were allowed to refine freely.

### Crystal data

**Table:**

(C_10_H_10_N_3_)_2_[ZnCl_4_]	*F*_000_ = 1120
*M**_r_* = 551.61	*D*_x_ = 1.536 Mg m^−^^3^
Monoclinic, *C*2/*c*	Mo *K*α radiation λ = 0.71073 Å
Hall symbol: -C 2yc	Cell parameters from 12557 reflections
*a* = 14.620 (3) Å	θ = 2.9–27.5º
*b* = 11.260 (2) Å	µ = 1.50 mm^−^^1^
*c* = 14.765 (3) Å	*T* = 150 (2) K
β = 101.13 (3)º	Prism, yellow
*V* = 2384.9 (8) Å^3^	0.1 × 0.1 × 0.1 mm
*Z* = 4	

### Data collection

**Table:**

Siemens SMART CCD area-detector diffractometer	2718 independent reflections
Radiation source: fine-focus sealed tube	2063 reflections with *I* > 2σ(*I*)
Monochromator: graphite	*R*_int_ = 0.029
*T* = 298(2) K	θ_max_ = 27.5º
phi and ω scans	θ_min_ = 3.6º
Absorption correction: multi-scan(SADABS in SAINT-NT; Bruker, 1999)	*h* = −18→18
*T*_min_ = 0.861, *T*_max_ = 0.861	*k* = −14→14
8907 measured reflections	*l* = −18→19

### Refinement

**Table:**

Refinement on *F*^2^	Secondary atom site location: difference Fourier map
Least-squares matrix: full	Hydrogen site location: inferred from neighbouring sites
*R*[*F*^2^ > 2σ(*F*^2^)] = 0.031	H atoms treated by a mixture of independent and constrained refinement
*wR*(*F*^2^) = 0.079	*w* = 1/[σ^2^(*F*_o_^2^) + (0.0297*P*)^2^ + 1.3722*P*] where *P* = (*F*_o_^2^ + 2*F*_c_^2^)/3
*S* = 1.17	(Δ/σ)_max_ = 0.001
2718 reflections	Δρ_max_ = 0.53 e Å^−^^3^
145 parameters	Δρ_min_ = −0.31 e Å^−^^3^
Primary atom site location: structure-invariant direct methods	Extinction correction: none

### Special details

**Table:**

Geometry. All e.s.d.'s (except the e.s.d. in the dihedral angle between two l.s. planes) are estimated using the full covariance matrix. The cell e.s.d.'s are taken into account individually in the estimation of e.s.d.'s in distances, angles and torsion angles; correlations between e.s.d.'s in cell parameters are only used when they are defined by crystal symmetry. An approximate (isotropic) treatment of cell e.s.d.'s is used for estimating e.s.d.'s involving l.s. planes.Least-squares planes (*x*,*y*,*z* in crystal coordinates) and deviations from them (* indicates atom used to define plane)- 7.5257 (0.0143) *x* - 6.4929 (0.0111) *y* + 10.6583 (0.0124) *z* = 0.0740 (0.0091)* -0.0004 (0.0017) N3 * 0.0057 (0.0017) C6 * -0.0049 (0.0019) C7 * -0.0009 (0.0021) C8 * 0.0062 (0.0022) C9 * -0.0057 (0.0021) C10Rms deviation of fitted atoms = 0.0046- 8.0734 (0.0131) *x* - 5.2989 (0.0109) *y* + 11.5436 (0.0110) *z* = 0.7176 (0.0115)Angle to previous plane (with approximate e.s.d.) = 7.15 (0.16)* 0.0093 (0.0015) N1 * 0.0017 (0.0017) C1 * -0.0085 (0.0019) C2 * 0.0045 (0.0020) C3 * 0.0061 (0.0019) C4 * -0.0132 (0.0016) C5Rms deviation of fitted atoms = 0.0081
Refinement. Refinement of *F*^2^ against ALL reflections. The weighted *R*-factor *wR* and goodness of fit *S* are based on *F*^2^, conventional *R*-factors *R* are based on *F*, with *F* set to zero for negative *F*^2^. The threshold expression of *F*^2^ > σ(*F*^2^) is used only for calculating *R*-factors(gt) *etc*. and is not relevant to the choice of reflections for refinement. *R*-factors based on *F*^2^ are statistically about twice as large as those based on *F*, and *R*- factors based on ALL data will be even larger.

### Fractional atomic coordinates and isotropic or equivalent isotropic displacement parameters (Å^2^)

**Table:**

	*x*	*y*	*z*	*U*_iso_*/*U*_eq_	Occ. (<1)
Zn	0.5000	0.81308 (3)	0.7500	0.03912 (11)	
Cl1	0.42368 (4)	0.92732 (5)	0.84063 (4)	0.05165 (16)	
Cl2	0.39576 (4)	0.69311 (5)	0.65703 (4)	0.05397 (16)	
N1	0.49641 (13)	0.42148 (17)	0.60363 (12)	0.0447 (4)	
H1N	0.437 (3)	0.478 (3)	0.5901 (7)	0.054*	0.50
C1	0.57114 (16)	0.4524 (2)	0.66942 (15)	0.0479 (5)	
H1	0.5699	0.5234	0.7013	0.057*	
C2	0.64808 (18)	0.3812 (3)	0.68967 (17)	0.0575 (6)	
H2	0.6992	0.4034	0.7343	0.069*	
C3	0.64815 (19)	0.2743 (3)	0.64175 (18)	0.0645 (7)	
H3	0.6995	0.2241	0.6552	0.077*	
C4	0.57324 (19)	0.2427 (2)	0.57501 (17)	0.0568 (6)	
H4	0.5732	0.1712	0.5434	0.068*	
C5	0.49701 (15)	0.3193 (2)	0.55522 (15)	0.0428 (5)	
N2	0.42142 (14)	0.29509 (19)	0.48707 (14)	0.0515 (5)	
H2N	0.4271 (2)	0.228 (2)	0.4519 (12)	0.062*	
C6	0.33748 (15)	0.3559 (2)	0.46259 (15)	0.0435 (5)	
C7	0.26855 (17)	0.3177 (2)	0.38963 (17)	0.0533 (6)	
H7	0.2780	0.2514	0.3550	0.064*	
C8	0.18643 (18)	0.3801 (3)	0.37007 (18)	0.0602 (7)	
H8	0.1393	0.3559	0.3219	0.072*	
C9	0.17357 (19)	0.4798 (3)	0.42241 (19)	0.0640 (7)	
H9	0.1180	0.5224	0.4103	0.077*	
C10	0.24470 (17)	0.5133 (3)	0.49185 (19)	0.0612 (7)	
H10	0.2370	0.5805	0.5262	0.073*	
N3	0.32622 (14)	0.45227 (19)	0.51276 (14)	0.0508 (5)	
H3N	0.378 (3)	0.4795 (17)	0.566 (3)	0.061*	0.50

### Atomic displacement parameters (Å^2^)

**Table:**

	*U*^11^	*U*^22^	*U*^33^	*U*^12^	*U*^13^	*U*^23^
Zn	0.0415 (2)	0.03647 (19)	0.03747 (19)	0.000	0.00295 (13)	0.000
Cl1	0.0604 (4)	0.0447 (3)	0.0527 (3)	0.0036 (3)	0.0181 (3)	−0.0025 (3)
Cl2	0.0527 (3)	0.0485 (3)	0.0531 (3)	−0.0037 (3)	−0.0089 (3)	−0.0057 (3)
N1	0.0472 (10)	0.0473 (11)	0.0400 (10)	0.0084 (9)	0.0093 (8)	0.0012 (8)
C1	0.0519 (13)	0.0508 (13)	0.0397 (12)	0.0013 (11)	0.0059 (10)	−0.0005 (10)
C2	0.0503 (14)	0.0758 (18)	0.0438 (13)	0.0054 (13)	0.0025 (10)	0.0067 (13)
C3	0.0570 (15)	0.0811 (19)	0.0537 (15)	0.0292 (14)	0.0068 (12)	0.0073 (14)
C4	0.0635 (16)	0.0573 (15)	0.0494 (14)	0.0217 (13)	0.0103 (12)	−0.0014 (12)
C5	0.0450 (11)	0.0492 (12)	0.0357 (10)	0.0077 (10)	0.0114 (9)	0.0059 (10)
N2	0.0539 (12)	0.0482 (12)	0.0517 (12)	0.0065 (9)	0.0089 (9)	−0.0097 (9)
C6	0.0437 (12)	0.0453 (12)	0.0427 (12)	0.0040 (10)	0.0111 (9)	0.0040 (10)
C7	0.0550 (14)	0.0547 (14)	0.0486 (13)	0.0022 (12)	0.0062 (11)	−0.0063 (12)
C8	0.0507 (14)	0.0729 (18)	0.0527 (14)	0.0006 (13)	−0.0006 (11)	−0.0021 (13)
C9	0.0477 (14)	0.0779 (19)	0.0627 (16)	0.0164 (13)	0.0016 (12)	−0.0026 (14)
C10	0.0524 (14)	0.0685 (17)	0.0607 (16)	0.0195 (13)	0.0061 (12)	−0.0118 (14)
N3	0.0457 (11)	0.0574 (12)	0.0478 (11)	0.0096 (9)	0.0050 (9)	−0.0066 (10)

### Geometric parameters (Å, °)

**Table:**

Zn—Cl2	2.2856 (8)	C5—N2	1.371 (3)
Zn—Cl2^i^	2.2856 (8)	N2—C6	1.391 (3)
Zn—Cl1^i^	2.2949 (7)	N2—H2N	0.9333
Zn—Cl1	2.2949 (7)	C6—N3	1.342 (3)
N1—C5	1.355 (3)	C6—C7	1.394 (3)
N1—C1	1.360 (3)	C7—C8	1.373 (4)
N1—H1N	1.0584	C7—H7	0.9300
C1—C2	1.366 (3)	C8—C9	1.396 (4)
C1—H1	0.9300	C8—H8	0.9300
C2—C3	1.397 (4)	C9—C10	1.365 (4)
C2—H2	0.9300	C9—H9	0.9300
C3—C4	1.371 (4)	C10—N3	1.359 (3)
C3—H3	0.9300	C10—H10	0.9300
C4—C5	1.395 (3)	N3—H3N	1.0328
C4—H4	0.9300		
			
Cl2—Zn—Cl2^i^	107.54 (4)	N2—C5—C4	121.9 (2)
Cl2—Zn—Cl1^i^	108.90 (3)	C5—N2—C6	129.7 (2)
Cl2^i^—Zn—Cl1^i^	109.79 (3)	C5—N2—H2N	115.2
Cl2—Zn—Cl1	109.79 (3)	C6—N2—H2N	115.2
Cl2^i^—Zn—Cl1	108.90 (3)	N3—C6—N2	116.8 (2)
Cl1^i^—Zn—Cl1	111.82 (4)	N3—C6—C7	121.9 (2)
C5—N1—C1	120.5 (2)	N2—C6—C7	121.3 (2)
C5—N1—H1N	119.7	C8—C7—C6	118.5 (2)
C1—N1—H1N	119.7	C8—C7—H7	120.8
N1—C1—C2	121.4 (2)	C6—C7—H7	120.8
N1—C1—H1	119.3	C7—C8—C9	120.1 (2)
C2—C1—H1	119.3	C7—C8—H8	120.0
C1—C2—C3	118.4 (2)	C9—C8—H8	120.0
C1—C2—H2	120.8	C10—C9—C8	118.2 (2)
C3—C2—H2	120.8	C10—C9—H9	120.9
C4—C3—C2	120.6 (2)	C8—C9—H9	120.9
C4—C3—H3	119.7	N3—C10—C9	122.6 (2)
C2—C3—H3	119.7	N3—C10—H10	118.7
C3—C4—C5	119.0 (2)	C9—C10—H10	118.7
C3—C4—H4	120.5	C6—N3—C10	118.6 (2)
C5—C4—H4	120.5	C6—N3—H3N	120.7
N1—C5—N2	118.03 (19)	C10—N3—H3N	120.7
N1—C5—C4	120.1 (2)		
			
C5—N1—C1—C2	−1.0 (3)	C5—N2—C6—N3	−1.0 (4)
N1—C1—C2—C3	−0.7 (4)	C5—N2—C6—C7	179.3 (2)
C1—C2—C3—C4	1.0 (4)	N3—C6—C7—C8	−1.0 (4)
C2—C3—C4—C5	0.4 (4)	N2—C6—C7—C8	178.7 (2)
C1—N1—C5—N2	−177.3 (2)	C6—C7—C8—C9	0.4 (4)
C1—N1—C5—C4	2.4 (3)	C7—C8—C9—C10	0.7 (4)
C3—C4—C5—N1	−2.1 (4)	C8—C9—C10—N3	−1.2 (5)
C3—C4—C5—N2	177.6 (2)	N2—C6—N3—C10	−179.1 (2)
N1—C5—N2—C6	−5.5 (4)	C7—C6—N3—C10	0.6 (4)
C4—C5—N2—C6	174.7 (2)	C9—C10—N3—C6	0.6 (4)

Symmetry codes: (i) −*x*+1, *y*, −*z*+3/2.

### Hydrogen-bond geometry (Å, °)

**Table:**

*D*—H···*A*	*D*—H	H···*A*	*D*···*A*	*D*—H···*A*
N1—H1N···N3	1.06	1.82	2.612 (3)	129
N3—H3N···N1	1.03	1.83	2.612 (3)	130
N1—H1N···Cl2	1.06	2.73	3.548 (2)	134
N3—H3N···Cl2	1.03	2.74	3.477 (3)	129
N2—H2N···Cl1^ii^	0.93	2.39	3.313 (3)	170

Symmetry codes: (ii) *x*, −*y*+1, *z*−1/2.
